# Supplementary material for: Nucleus pulposus cell network modelling in the intervertebral disc
Source: NPJ Syst Biol Appl. 2025 Jan 31;11:13. doi: 10.1038/s41540-024-00479-6 (PMC11785752; doi:10.1038/s41540-024-00479-6)
Supplement: Supplementary file 1 — Supplementary information [file 41540_2024_479_MOESM1_ESM.pdf]

Nucleus Pulposus Cell Network Modelling in the Intervertebral Disc

Sofia Tseranidou*[1], Maria Segarra-Queralt[1], Francis Kiptengwer Chemorion[1],Christine Le Maitre[2], Janet Piñero[3], Jérôme Noailly[1]

1. Department of Engineering, Universitat Pompeu Fabra, Spain; 2. Department of Oncology and Metabolism, University of Sheffield, United Kingdom; 3. IMIM, Spain

SUPPLEMENTARY MATERIAL

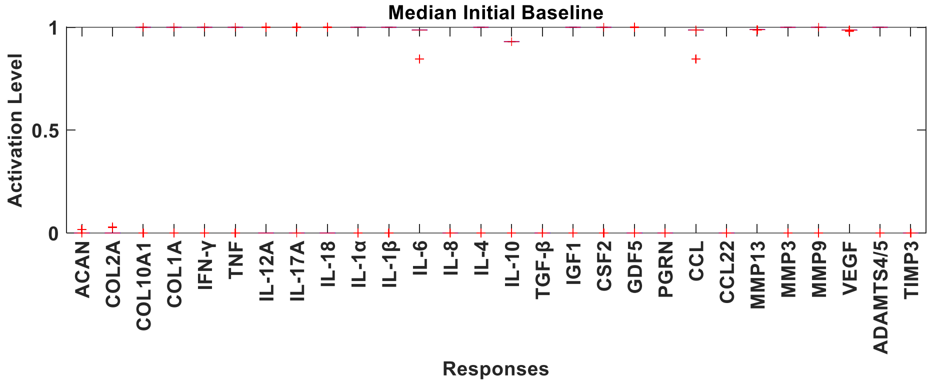

Fig.1. Boxplots of the initial RNM network. The baseline of the initial regulatory network model (RNM) for every protein is represented by red boxplots.

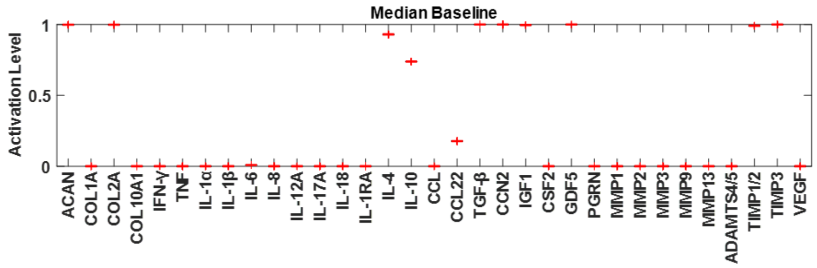

Fig.2. Boxplots of the initial RNM network. The baseline of the enriched regulatory network model (RNM) for every protein is represented by red boxplots.

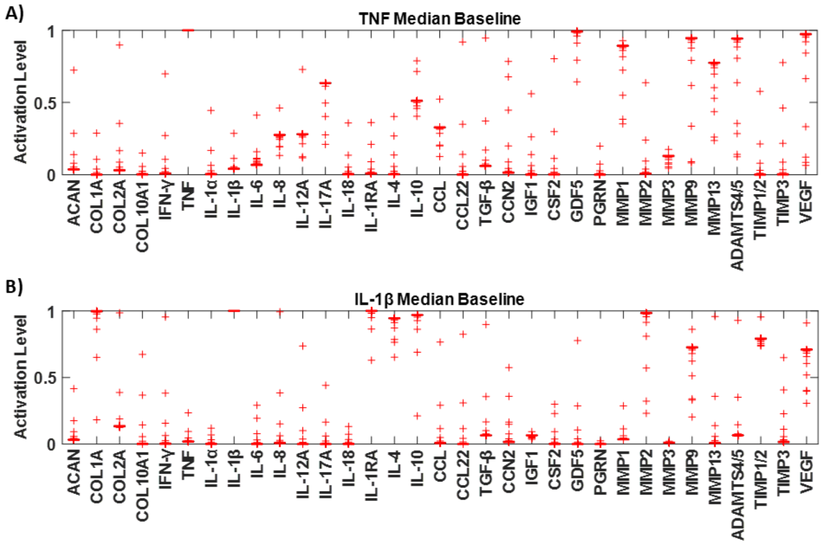

Fig.3. Boxplots of the perturbed baselines. The baseline of the enriched regulatory network model (RNM) for every protein after A) TNF stimulation and B) IL-1β stimulation is represented by red boxplots.

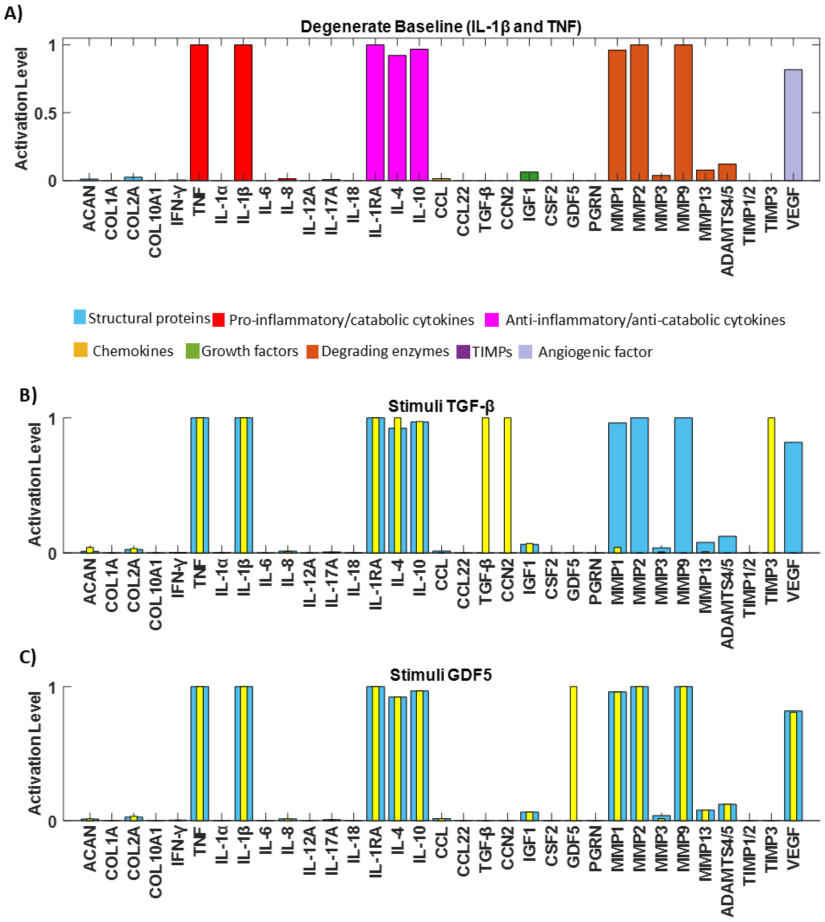

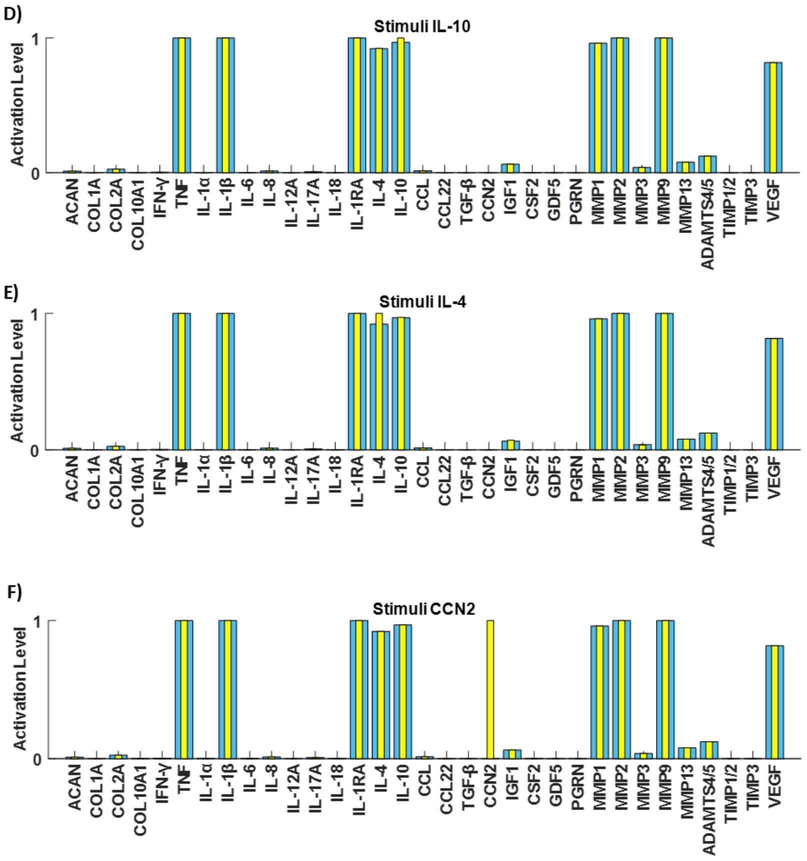

Fig.4. Assessment of the network through independent tests. A degenerate baseline was produced to promote catabolism by clamping TNF and IL-1β (Fig.3A) to 1. Rescue strategies were simulated by stimulating the degenerate baseline with B) TGF-β, C) GDF5, D) IL-10, E) IL-4 and F) CCN2 (yellow bars).

**Nodes**	**Activators**	**Inhibitors**
ACAN	GDF5,TGF-β	IL-18, IL-1β, IL-17A, TNF
COL2A	GDF5	IL-18, IL-1β, TNF, IL-17A
COL10A1	IL-1β	NOTHING
COL1A	IL-1β	IL-17A
IFN-γ	IL-1β	NOTHING
TNF	IL-1β	TIMP3
IL-12A	NOTHING	IL-1β,IL-4
IL-17A	NOTHING	IL-1β
IL-18	NOTHING	IL-1β
IL-1α	IL-1β	NOTHING
IL-1β	IL-1β	NOTHING
IL-6	IL-1β,IL-4,IL-17A	NOTHING
IL-8	IL-1β	IL-4
IL-4	IL-1β	NOTHING
IL-10	IL-1β,PGRN	NOTHING
TGF-β	NOTHING	NOTHING

IGF1	IL-1β	NOTHING
CSF2	IL-1β	NOTHING
GDF5	NOTHING	IL-1β
PGRN	NOTHING	NOTHING
CCL	IL-1β,IL-17A,TNF	NOTHING
CCL22	IL-4	IL-1β
MMP13	IL-18, IL-1β, IL-6, TNF, IL-17A	NOTHING
MMP3	IL-1β,IL-6,TNF	GDF5
MMP9	TNF	NOTHING
VEGF	IL-17A,IL-1β,IL-6	TIMP3
ADAMTS4/5	IL-1β,TNF	NOTHING
TIMP3	NOTHING	NOTHING

Table 1: Initial topology. Summary of interactions in the IVD knowledge-based regulatory network model derived from the literature via PubMed.

**Nodes**	**Activators**	**Inhibitors**
ACAN	GDF5,TGF-β,IL-4,IL-10,IGF1	IL-18,IL-1β,IL-17A,TNF,IL-6,MMP2
COL1A	IL-1β	TNF
COL2A	GDF5,TGF-β,IL-10,IL-4,IGF1	IL-1β,TNF,IL-17A,IL-18,IL-6
COL10A1	IL-1β	IL-10
IFN-γ	IL-1β	IL-4
TNF	IL-1β,IL-17A,IL-6	TIMP3,IL-10,IL-4,TGF-β,GDF5
IL-1α	IL-1β	IL-10,IL-4
IL-1β	IL-1β,IL-17A,TNF	IL-10,IL-4,TGF-β,GDF5
IL-6	IL-1β,IL-17A,TNF,IL-18,IL-6,IL-8,IL-4	IL-10
IL-8	IL-1β,TNF,IL-17A	IL-4,IL-10,IL-6
IL-12A	IL-17A	IL-4,IL-1β,IL-10
IL-17A	TNF	IL-1β,IL-4,IL-10,PGRN
IL-18	IL-1β	IL-10,IL-4
IL-1RA	IL-1β	NOTHING
IL-4	IL-1β,TGF-β	IL-17A
IL-10	IL-1β,PGRN,IL-17A,IL-4	NOTHING
CCL	IL-1β,IL-17A,TNF	IL-10,IL-4,TGF-β
CCL22	IL-4	IL-1β,IL-10
TGF-β	NOTHING	TNF,IL-1β
CCN2	TGF-β	NOTHING
IGF1	IL-4	IL-1β,IL-6

CSF2	IL-1β	IL-10
GDF5	NOTHING	IL-1β
PGRN	NOTHING	NOTHING
MMP1	IL-1β,TNF,IL-17A,IL-18,MMP3	TIMP1/2,TGF-β
MMP2	IL-1β, TIMP1/2	TGF-β, IGF1
MMP3	IL-1β,TNF,IL-6,IL-17A,IL-18	GDF5,TIMP1/2,IGF1,TGF-β,IL-4,TIMP3,IL-1RA,CCN2
MMP9	TNF,MMP2,MMP1	TGF-β
MMP13	TNF,IL-1β,IL-6,IL-18,IL-8,IFN-γ	TGF-β,TIMP1/2,IL-1RA,CCN2
ADAMTS4/5	TNF,IL-1β,IL-6,VEGF	TGF-β,TIMP3,IL-1RA
TIMP1/2	IL-6,IL-10,TGF-β,IGF1	TNF
TIMP3	TGF-β	IL-17A
VEGF	IL-1β,IL-6,IL-17A,MMP3,ADAMTS4/5,MMP13	TIMP3

Table 2: Enriched topology. Summary of interactions in the IVD knowledge-based regulatory network model derived from the literature via PubMed enriched by using the STRING database and manual enrichment via PubMed.
